# SNARE Protein Syntaxin-1 Colocalizes Closely with NMDA Receptor Subunit NR2B in Postsynaptic Spines in the Hippocampus

**DOI:** 10.3389/fnmol.2016.00010

**Published:** 2016-02-05

**Authors:** Suleman Hussain, Håvard Ringsevjen, Daniel L. Egbenya, Torstein L. Skjervold, Svend Davanger

**Affiliations:** Division of Anatomy, Department of Molecular Medicine, Institute of Basic Medical Science, University of OsloOslo, Norway

**Keywords:** SNARE, synapse, vesicles, hippocampus, PSD, active zone, electron microscopy, NMDA

## Abstract

Syntaxins are a family of membrane-integrated proteins that are instrumental in exocytosis of vesicles. Syntaxin-1 is an essential component of the presynaptic exocytotic fusion machinery in the brain and interacts with several other proteins. Syntaxin-1 forms a four-helical bundle complex with proteins SNAP-25 and VAMP2 that drives fusion of vesicles with the plasma membrane in the active zone (AZ). Little is known, however, about the ultrastructural localization of syntaxin-1 at the synapse. We have analyzed the intrasynaptic expression of syntaxin-1 in glutamatergic hippocampal synapses in detail by using quantitative postembedding immunogold labeling. Syntaxin-1 was present in highest concentrations at the presynaptic AZ, supporting its role in transmitter release. Presynaptic plasma membrane lateral to the AZ, as well as presynaptic cytoplasmic (PreCy) vesicles were also labeled. However, syntaxin-1 was also significantly expressed in postsynaptic spines, where it was localized at the postsynaptic density (PSD), at postsynaptic lateral membranes and in postsynaptic cytoplasm. Postsynaptically, syntaxin-1 colocalized in the nanometer range with the N-methyl-D-aspartate (NMDA) receptor subunit NR2B, but only weakly with the AMPA receptor subunits GluA2/3. This observation points to the possibility that syntaxin-1 may be involved with NR2B vesicular trafficking from cytoplasmic stores to the postsynaptic plasma membrane, thus facilitating synaptic plasticity. Confocal immunofluorescence double labeling with PSD-95 and ultrastructural fractionation of synaptosomes also confirm localization of syntaxin-1 at the PSD.

## Introduction

Individuals adapt to changes in the environment through synaptic plasticity. Synapses are junctions between neurons where the flow of information can be modified (Pozo and Goda, 2010). The amino acid glutamate is the predominant excitatory neurotransmitter in the brain (Fonnum, 1984). Glutamatergic transmission mainly occurs through activation of postsynaptic receptors of the AMPA (α-Amino-3-hydroxy-5-methyl-4-isoxazolepropionic acid) class (Rosenmund et al., 1998). Common forms of synaptic plasticity involves vesicular insertion or retraction of AMPA receptors in the postsynaptic plasma membrane, regulated by calcium influx through glutamate receptors of the NMDA (N-methyl-D-aspartate) class, which also seem to be inserted in the synapse through vesicular mechanisms (Lu et al., 2001; Hanley, 2008). We have recently shown, for the first time, the presence of small, cytoplasmic vesicles in postsynaptic spines (Hussain and Davanger, 2015). The existence of such vesicles is a necessary condition for vesicular regulation of synaptic strength.

Trafficking of vesicles in nerve cells, including exocytosis of neurotransmitter, is dependent on accurate and specific target membrane recognition, attachment, and fusion (Bajjalieh and Scheller, 1995). The molecular fusion machinery (soluble N-ethylmaleimide-sensitive factor attachment protein receptor complex, or SNARE complex) consists of integral membrane proteins on the vesicle and target membranes that interact with each other during vesicle docking and fusion (Söllner et al., 1993). A key step is the assembly of the exocytotic core complex. In presynaptic nerve terminals, it consists of VAMP2 (also known as synaptobrevin), which is an integral synaptic vesicle protein, and syntaxin-1 and SNAP-25 at the plasma membrane (Hayashi et al., 1994; Jahn and Südhof, 1994).

Several members of the mammalian syntaxin family have been identified. Syntaxins 1, 2, 3 and 4 were the first group of syntaxins discovered. These proteins are predominantly localized at the plasma membrane and mediate constitutive and regulated vesicle transport events at the cell surface (Inoue et al., 1992; Bennett et al., 1993; Gaisano et al., 1996; Low et al., 1996). Syntaxin-1 comprises two isoforms, A and B, which both are nervous system-specific proteins implicated in the docking of synaptic vesicles with the presynaptic plasma membrane. They share 84% amino acid identity. Syntaxin-1A is composed of 288 amino acids. The protein is most commonly found in neurons and neuroendocrine cells making up approximately 1% of the total amount of brain proteins (Lang and Jahn, 2008).

In the cerebellum, syntaxin-1 has been detected at high densities on the interior of the synaptosomal plasma membranes and on presynaptic vesicles (Koh et al., 1993). Apart from that, we know little about the ultrastructural localization of this SNARE protein within synapses. The common assumption is that in synapses, SNARE proteins are expressed in the presynaptic terminal. Recent reports, however, indicate that some SNARE isoforms have postsynaptic functions. Syntaxin-3 and SNAP-47 are expressed in postsynaptic elements and seem to be involved in exocytosis of AMPARs during LTP (Jurado et al., 2013). VAMP2 is also expressed in postsynaptic spines where it plays a role in trafficking of the GluA1 AMPA receptor subunit (Hussain and Davanger, 2015).

We hypothesized that syntaxin-1 may be a candidate for postsynaptic vesicle trafficking, corresponding to its presynaptic function. Despite the number of studies describing SNARE protein structure and function in general, there is limited information about the intrasynaptic localization of syntaxin-1. In the present study, we have used quantitative postembedding immunocytochemistry on ultrathin sections of the hippocampus to determine subsynaptic localizations of syntaxin-1 in Schaffer collateral synapses in the CA1 area. This enabled us to perform a detailed ultrastructural analysis of syntaxin-1 localization in subsynaptic compartments, specifically focusing on the PSD and spine cytoplasm.

## Materials and Methods

### Antibodies

Anti-syntaxin-1 (Alomone Labs, Jerusalem, Israel Cat#ANR-002) was used at 1:40,000, anti-β-tubulin (Tuj1) (Covance, CA, USA, Cat#MMS-435P) at 1:10,000, anti-PSD-95 (Abcam, Cambridge, UK, Cat#ab13552) at 1:10,000 and anti-synaptophysin (Millipore, Darmstadt, Germany, Cat#MAB368) at 1:5000 for western blotting. Anti-syntaxin-1 (Millipore, MA, USA, Cat#AB5820; Bennett et al., 1992) and anti-syntaxin-1 (Sigma, Missouri, USA, Cat#S; Besalduch et al., 2010) were used at 1:10–1:50, GluA2&3 (Millipore, MA, USA, Cat#AB1506; Volz et al., 2011) at 1:30 and anti-NR2B (Abcam, Cambridge, UK, Cat#ab65783; Nath et al., 2014) at 1:400 for postembedding immunogold electron microscopy. Anti-syntaxin-1 (Alomone Labs, Jerusalem, Israel, Cat#ANR-002) was used at 1:200 for light microscopy. Anti-syntaxin-1 (Millipore, Massachusetts, USA, Cat#AB5820) was used at 1:100, anti-TUJ 1 (Covance, CA, USA, Cat#MMS-435P) at 1:100, anti-synaptophysin (Abcam, Cambridge, UK, Cat#ab180008) at 1:100 and anti-PSD-95 (Novus Biologics, ON, Canada, Cat#NB300-556) at 1:100 for immunofluorescence confocal microscopy. Secondary antibodies: Donkey anti-rabbit Cy3 (Jackson Immuno, MD, USA, Cat#711-165-152) and donkey anti-mouse A488 (Invitrogen, CA, USA, Cat#A21202) were used at 1:1000 for immunofluorescence confocal microscopy. Mouse anti-rabbit alkaline phosphatase (Sigma, MO, USA, Cat#A3687) was used at 1:10,000 for western blotting. IgG coupled to 10 or 20 nm colloidal gold (British BioCell International, Cardiff, UK, Cat#R14007) and (Abcam, Cambridge, UK, Cat#ab27234) was used at 1:20 for electron microscopy. Biotynilated goat anti-rabbit (Abcam, Cambridge, UK, Cat#Ab64256) was used at 1:100 together with a streptavidin biotynilated horseradish peroxidase complex (GE Healthcare, Buckinghamshire, UK, Cat#RPN1051V) at 1:100 for light microscopy.

### Animals

All animal experimentation was carried out in accordance with the European Communities Council Directive of 24 November 1986 (86/609/EEC). Formal approval to conduct the experiments described was obtained from the animal subjects review board of the Norwegian Governmental Institute of Public Health (Oslo, Norway). Care was taken to minimize the number of animals used and to avoid suffering. Wistar rats (from Scanbur, Nittedal, Norway) weighing 250–300 g were used for electron microscopy, western blot and light microscopy. Wistar Kyoto rats from Harlan Laboratories, Horst, Netherlands, (males), weighing about 100 g were used for preparation of active zone and post synaptic density fraction. One to five days old Wistar rats of either sex were used for primary hippocampal cultures.

### Preparation of Synaptosome and Vesicle Membrane Fractions

Ten rats were decapitated, the brains were dissected out and submerged in ice cold Hepes-buffered sucrose (0.32 M sucrose, 4 mM Hepes, pH 7.4) containing a protease inhibitor cocktail (Promega, Winconsin, USA). The tissue was homogenized in Hepes buffer with a glass-Teflon homogenizer (900 rpm, 10–15 strokes) and centrifuged (800–1000 g, 10 min, 4°C). The postnuclear supernatant, S1, was centrifuged (10,000 g, 15 min), and the pellet containing crude synaptosomes was resuspended in 10 volumes of Hepes-buffered sucrose and centrifuged (10,000 g, 15 min). The synaptosomal fraction was resuspended in 10 mM sucrose, centrifuged (161,000 g, 25 min), the gradient interphase was collected and diluted in Hepes-buffered sucrose, and centrifuged again (161,000 g, 25 min) to get a pellet containing pure synaptosomes. To extract synaptic vesicles, the pure synaptosome pellet was lysed by hypo-osmotic shock in nine volumes of ice cold H_2_O plus protease/phosphatase inhibitors and three strokes with a glass-Teflon homogenizer, this solution was adjusted to 4 mM Hepes and mixed at 4°C for 30 min to ensure complete lysis. The lysate was centrifuged (161,000 g, 25 min), before the resulting supernatant again was centrifuged (165,000 g for 2 h) and then resuspended in 50 mM Hepes, 2 mM EDTA plus protease/phosphatise inhibitors.

### Preparation of Active Zone and PSD Fraction

Four rats were sacrificed and their hippocampi were dissected out and submerged in ice-cold Hepes buffered sucrose (0.32 M sucrose, 4 mM Hepes, pH 7.4) containing a protease inhibitor tablet cocktail (Roche, Indianapolis, USA). Pairs of the eight hippocampi were homogenized in a glass-Teflon homogenizer and centrifuged (800–1000 g, 10 min, 4°C) in 2 ml to remove nuclei and cellular debris. The postnuclear supernatant, S1, was centrifuged and washed as described earlier (2× 10,000 g, 15 min) in Hepes buffered sucrose (0.32 M sucrose, 4 mM Hepes, pH 7.4). The washed synaptosomal pellet was resuspended in 1ml Hepes buffered sucrose, combined two and two, and carefully laid onto 1ml 0, 8 M sucrose containing a protease inhibitor tablet cocktail and centrifuged (230,000 g, 15 min, 4°C). The resulting pellet contained pure synaptosomes.

The further steps are previously described in the literature (Phillips et al., 2001) but have been slightly modified for our purposes. The pure synaptosomal pellet was diluted 10 volumes in 0.1 mM CaCl_2,_ and then lysed by hypo-osmotic shock and 1% Triton X-100 in 10 volumes of 2× lysis buffer (8 mM Hepes, 2% Triton X-100, complete protease inhibitor tablet cocktail, pH 6, 0) and incubated (30 min, 4°C) before centrifuged (40,000 g, 30 min, 4°C). Non-synaptic proteins from the supernatant were decanted and proteins precipitated with 10 volumes acetone at −20°C and recovered by centrifugation (16,000 g, 30 min, 4°C), and later on saved for western blotting in Hepes buffer with 0.2% SDS. The pellet containing pH-stable synapses was incubated (30 min, 4°C) in 1ml PSD-buffer (4 mM Trizma base, 1% Triton X-100, complete protease inhibitor tablet cocktail, pH 8.0) and centrifuged as above. The diluted synapses were separated into a supernatant containing the presynaptic AZ and a triton-insoluble PSD-pellet. The presynaptic supernatant was decanted and proteins precipitated with 10 volumes of acetone at −20°C and subsequently recovered by centrifugation (16,000 g, 30 min, 4°C). Both the precipitated supernatant and PSD-pellet were saved for western blotting in Hepes buffer with 0.2% SDS.

### Immunoblotting

The whole brain homogenate and membrane fractions from four adult Wistar rats were run on 4–20% SDS-acrylamide, electroblotted onto PVDF membranes (Hoefer Scientific Instruments, San Francisco, CA, USA) and immunostained with primary antibodies and horseradish peroxidase–linked secondary antibody. The signal was detected by fluorescence using ECF substrate (Amersham Biosciences, UK, Cat#45-000-947). The fluorescence signals were visualized by a fluorescence digital camera detection system (Typhoon and Kodak scanner). Band intensities were quantified by using adobe photoshop and normalized to loading control (B-tubulin).

### Bright Field Microscopic Studies

Free floating vibratome sections (50 μm) from rat brain were treated with 1 M ethanolamine-HCL (pH 7.4), blocked with 3% (v/v) normal calf serum (NCS) in 0.1 M sodium phosphate buffer, pH 7.4, and incubated with primary antibodies overnight at room (overnight/room temperature), followed by incubation with secondary antibodies for 1 h at room temperature and development with the biotin-streptavidin-peroxidase system and 3,3-Diaminobenzidine (DAB).

### Quantitative Postembedding Immunogold Electron Microscopy

#### Perfusion Fixation

For electron microscopy studies the rats were deeply anesthetized with Equithesin (0.4 ml/100 g body weight), followed by intracardiac perfusion with 10–15 s flush of 4% Dextran-T70 in 0.1 M sodium phosphate buffer (pH 7.4), before perfusion with a mixture of 4% formaldehyde and 0.1% glutaraldehyde in the same buffer. Time for perfusion was approximately 15 min. The rats were then left overnight in a cold room. The next day, the brains were carefully dissected out and stored in a sodium phosphate buffer (0, 1 M with 0.4% FA and 0.01% GA).

### Fixation of HELA Cells

HELA cell cultures were grown in DMDM with 10% fetal calf serum (Gibco BRL, Invitrogen) and 0.1% glutamine. The cells were plated to confluence and fixed with 4% FA and 0.1% GA. HELA cells were incubated 30 min in 10% glycerol, 30 min in 20% glycerol and overnight in 30% glycerol. The cells were centrifuged at 1000 g for 5 min.

### Electron Microscopy

Pellet of HELA cells and small (0.5–1.0 mm) blocks dissected out from colon and the CA1 area of the hippocampus were freeze substituted, sectioned, and immunolabeled essentially as described previously (Mathiisen et al., 2006). For double labeling, the sections were first incubated with either rabbit polyclonal anti-NR2B or anti-GluA2/3 followed by goat anti-rabbit coupled to 10 nm colloidal gold. The sections were exposed to formaldehyde vapor at 80°C for 1 h and thereafter incubated with rabbit polyclonal anti-syntaxin-1, followed by goat anti-rabbit coupled to 20 nm colloidal gold. The sections were the contrasted with uranyl acetate and lead citrate and examined with Fei Tecnai 12 electron microscope.

### Quantification and Statistical Analysis of Immunogold Labeling

Electron micrographs from rats (*n* = 3) were obtained from asymmetric synapses (Schaffer collateral synapses) from the middle layer of stratum radiatum of the CA1 region of the hippocampus. Syntaxin-1 immunolabeling was quantified as number of gold particles/μm of membrane length in asymmetric synapses and as number of gold particles/μm^2^ in the intracellular compartment. One hundred and eighty profiles from each region of interest were quantified (60 profiles from each rat). Specific plasma membrane and cytoplasmic compartments were defined and used for quantifications. They correspond to: the postsynaptic density (PSD); the active zone (AZ); the lateral/perisynaptic membranes, i.e., on each side of the PSD (PoL) or the AZ (PreL); the postsynaptic cytoplasm (PoCy); the presynaptic cytoplasm (PreCy); the dendritic plasma membrane (DM); the dendrite cytoplasm (DCy). The synaptic lateral membranes were defined for convenience of measurement as equal to the length of the PSD, on both sides of the PSD or AZ, for all synapses. Only synapses with clearly visible synaptic membranes and PSD were selected for quantitative analysis. An in-house extension to the analysis software connected with SPSS (SPSS Inc, Chicago, IL, USA) was used to quantify the gold particle labeling of the regions of interest. The software calculated area gold particle density (number per unit area) over cytoplasmic compartments and linear particle density (number per unit length of curve) over membrane domains. In the latter case, it measured the distance from each particle-center to the membrane and included only those particles, which were within an operator-defined distance from the curve segment. For plasma membranes the inclusion distance was symmetric between −21 nm, and +21 nm (negative signifying an intracellular location). Data for particles were collected in ASCII files as flat tables and exported to SPSS for further statistical and graphical analysis. Double immunogold labeling was quantified using imageJ. Gold particles located within 47 nm radius from each other were classified as colocalized. The distance 47 nm was determined by summarizing length of two times primary antibody (16 nm), length of two times secondary antibody (16 nm), radius of 10 nm gold particle (5 nm) and radius of 20 nm gold particle (10 nm).

### Preparation of Hippocampal Neuronal Cultures

Primary hippocampal cultures containing both neurons and glial cells of 1–5 day old rats (Wistar) were prepared essentially as previously described (Hasegawa et al., 2004). Briefly, cultures were prepared from 1–4 day old rats. They were maintained on glass coverslips in cell medium (Gibcos MEM with the addition of 30 mg/100 ml glutamine; 2.5 mg/100 ml insulin; 5–10% fetal calf serum; 2 ml/100 ml B-27 and 2–10 μl/100 ml ARA-C in 5% CO_2_/95% air incubator at 37°C. The cultures were used for experiments after 14–21 days. The sections were fixed in 4% formaldehyde and labeled with primary antibody in 2% (v/v) normal calf serum, 1% (w/v) bovine serum albumin, and 0.4% saponin in 0.1 M sodium phosphate buffer (overnight/room temperature). The sections were rinsed in 0.1 M sodium phosphate buffer, incubated for 30 min with secondary antibodies at room temperature and rinsed again in 0.1 M sodium phosphate buffer. The coverslips were mounted top down with Fluormont mounting media (Southern Biotech), and examined with an Axioplan 2 equipped with a LSM 5 Pascal scanner head (Carl Zeiss, Heidelberg, Germany).

## Results

### Syntaxin-1 is Abundant in Brain Areas with High Densities of Synapses

**Figure 1:**
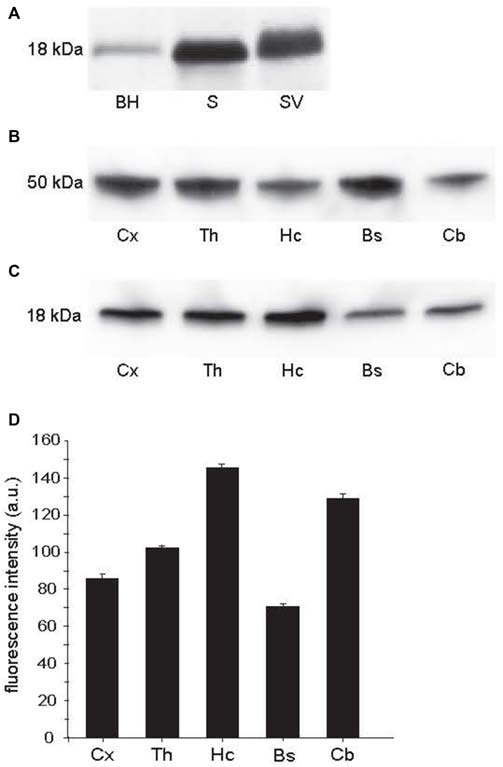
**Western blots from wild type rat brain. (A)** BH: brain homogenate; S: crude synaptosomes; SV: synaptic vesicles. Protein loaded 30 μg. **(B)** β-tubulin, used as loading control, in samples of homogenate from different brain regions. Protein loaded 20 μg. **(C)** Syntaxin-1 in homogenate samples from different brain regions, i.e., cortex (Cx, mean: 86, STD: 5.29, SEM: 2.64), thalamus (Th, mean: 104, STD: 2.3, SEM: 1.67), hippocampus (Hc, mean: 147, STD: 5.15, SEM: 2.57), brain stem (Bs, mean: 71, STD: 5.92, SEM: 2.96) and cerebellum (Cb, mean: 129, STD: 5.22, SEM: 2.61). Protein loaded 20 μg. **(D)** Quantitation of intensities of bands seen above, indicating highest concentration in the hippocampus, lowest in the brain stem. Error bars show SEM, based on four different blots.

To determine syntaxin-1 protein expression among different regions in the CNS, we performed western blotting with anti-syntaxin-1 antibody (Figure 1). The antibody gave a single band on crude whole brain homogenate, the corresponding band was much stronger on synaptosome and synaptic vesicle fractions (Figure 1A), as expected for a synaptic specific protein. The synaptic vesicle band indicates that the protein is present also on vesicles. Furthemore, syntaxin-1 was expressed in different brain regions (Figure 1C). The highest concentrations of syntaxin-1, normalized against β-tubulin (Figure 1B), were observed in the hippocampus, the lowest in the brain stem (Figure 1D). Thalamus and cortex showed respectively 30 and 41% less syntaxin-1 expression than the hippocampal level. Among the regions analyzed, the cerebellum showed the highest concentration of syntaxin-1 next to hippocampus.

### Syntaxin-1 Immunoreactivity in Neuronal Cells

**Figure 2:**
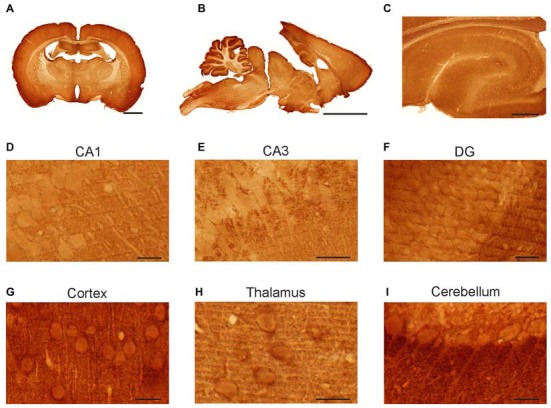
**Light micrographs of rat brain sections.** Vibratome sections immunolabeled for syntaxin-1. Immunoperoxidase staining. Anti-syntaxin-1 produced staining of neurons in hippocampus, cerebellum, cerebral cortex and thalamus **(A–C)**. In the hippocampus the somata and proximal dendrites of pyramidal neurons were moderate stained in sub-regions of CA1 **(D)** and CA3 **(E)**, as were the granule cells in dentate gyrus **(F)**. Strong immunostaining was found proximal dendrites of Purkinje cells in the cerebellar cortex **(I)**, and in the pyramidal cells of the cerebral cortex **(G)**. Immunolabeling was also observed in thalamic neurons **(H)**. Scale bar: **(A)** 2000 μm. **(B)** 5000 μm. **(C)** 500 μm. **(D,G)** 20 μm. **(F,H,I)**: 25 μm. **(E)** 50 μm.

Whole rat brain sections were immunostained with anti-syntaxin-1 antibody in order to determine protein expression in different brain regions and at cellular levels (Figure 2). The antibody gave distinct labeling of neuron rich areas. Specifically, the cerebral cortex, the cerebellar cortex and the hippocampus were heavily labeled (Figures 2A–C). White matter, e.g., the corpus callosum and cerebellar white matter, was only weakly stained. At the cellular level, moderately stained dendrites in several areas, e.g., in the hippocampus and cortex (Figures 2D,E,G) can be seen outlined with densely stained puncta, probably signifying presynaptic boutons. Specifically, hippocampal pyramidal neurons in CA1 (Figure 2D) and CA3 (Figure 2E), as well as cortical pyramidal neurons (Figure 2G), showed moderate staining of the cell body cytoplasm and proximal dendrites. Granule cells in the dentate gyrus (Figure 2F) and cerebellum, as well as cerebellar Purkinje cells, were also immunopositive for syntaxin (Figures 2F,I). Thalamic neurons showed strong labeling of the cell body cytoplasm (Figure 2H).

### Syntaxin-1 is Expressed Postsynaptically

**Figure 3:**
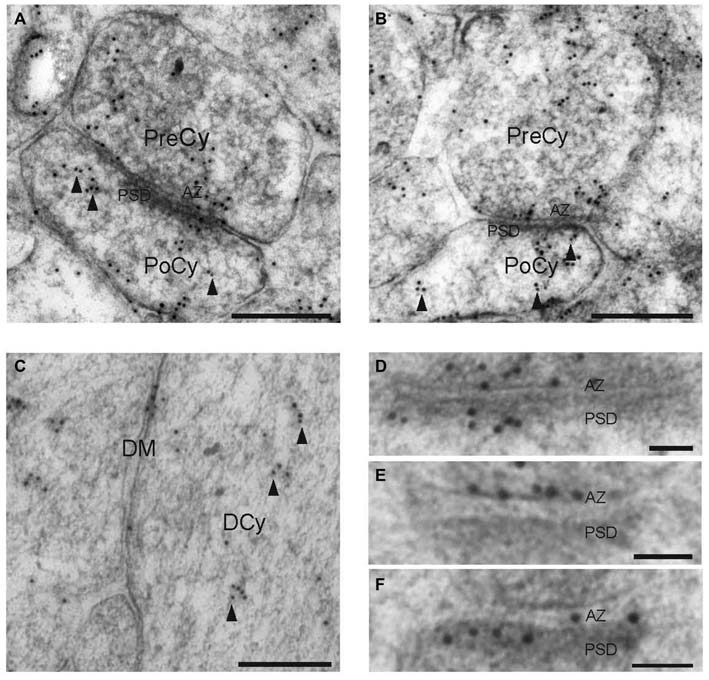
**Electron micrographs showing syntaxin-1 immunogold labeling of the synapses and dendrites in the CA1 region of the rat hippocampus. (A,B)** Gold particles are present over pre- and post-synaptic vesicles, as well as synaptic plasma membranes. **(C)** Immunogold labeling of syntaxin-1 in dendrite. **(D–F)** Gold particles located over active zone and post synaptic densities. Scale bars: **(A,B)** 250 nm. **(C)** 200 nm. **(D−F)** 50 nm.

In order to determine the localization of syntaxin-1 within synapses, we conducted high-resolution immunogold electron microscopical investigations of the protein in the hippocampal Schaffer collateral synapse. Figure 3 shows electron micrographs of syntaxin-1 immunogold labeling of glutametergic synapses in stratum radiatum of the hippocampal CA1 area. Presynaptically, the highest density of syntaxin-1 immunoreactivity in the presynaptic compartment was located at the AZ (Figures 3A,B,D,E), as would be expected from a t-SNARE. Many gold particles were also observed attached to presynaptic vesicles. However, postsynaptic spines were also clearly labeled, with immunogold labeling showing a somewhat similar labeling pattern as in the presynaptic terminal. Gold particles were noticably associated with the PSD (Figures 3D,F), but many postsynaptic vesicles were also immunopositive (Figures 3A,B). Some gold particles were also located along the pre- and postsynaptic lateral membranes. In dendrites, gold particles were associated with cytoplasmic vesicles along microtubules and attached to the DM (Figure 3C).

**Figure 4:**
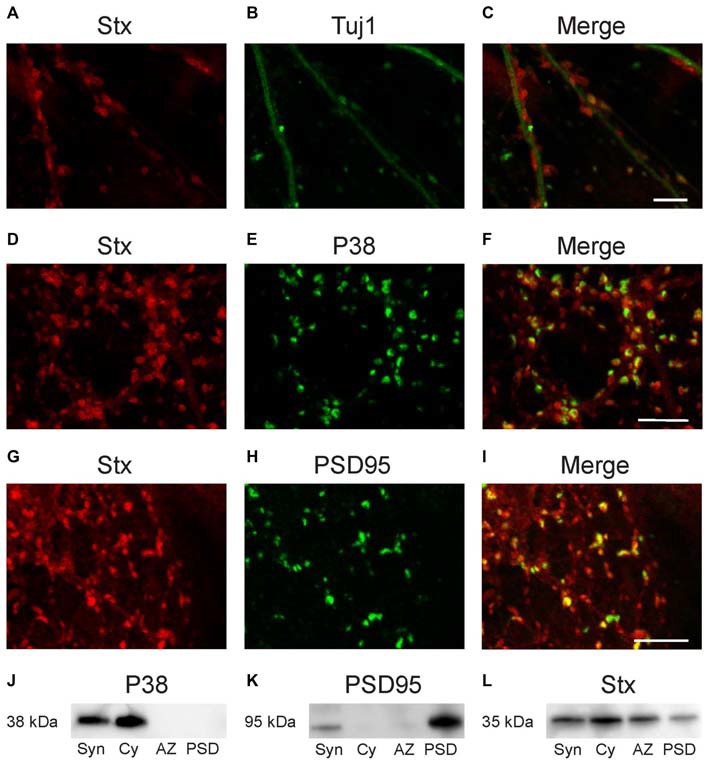
**Confocal images of dissociated hippocampal cultures.** Double labeling with anti-syntaxin-1 **(A)** and anti-Tuj1 **(B)** demonstrates that syntaxin-1 is located along the dendrites and gives characteristic punctate labeling **(C)**. Double labeling with antibodies against syntaxin-1 **(D)** and synaptophysin **(E)** indicates that syntaxin-1 is colocalized with presynaptic marker **(F)**. Double labeling for syntaxin-1 **(G)** and PSD-95 **(H)** shows partial colocalization postsynaptic **(I)**. Scale bars: 10 μm. **(J–L)** Western blot analysis of synaptosomes (Syn), synaptic cytosolic fraction (Cy), active zone (AZ) and post synaptic density (PSD). Labeled with anti-synaptophysin **(J)** anti-PSD95 **(K)** and anti-syntaxin-1 **(L)**.

Double immunofluorescence labeling of dissociated hippocampal cultures confirmed the synaptic localization of syntaxin-1. As is evident from Figure 4, syntaxin is co-expressed both with the PDS protein PSD-95 (Figures 4G–I) and the presynaptic vesicle protein synaptophysin (Figures 4D–F). We further isolated PSD, AZ and synaptic cytosolic fractions from brain homogenate. Western blot labeling of these fractions with anti-syntaxin-1 showed results consistent with the EM data (Figures 4J–L). The protein is clearly expressed in both presynaptic AZ and in the PSD fraction, and is also present in synaptic cytosol (Figure 4L). Control immunostaining with antibodies against specific pre- or postsynaptic marker proteins synaptophysin (P38) and PSD95 show that the AZ and PSD fractions were not contaminated with each other (Figures 4J,K).

### Relative Concentrations of Syntaxin-1 in Synaptic Subregions

**Figure 5:**
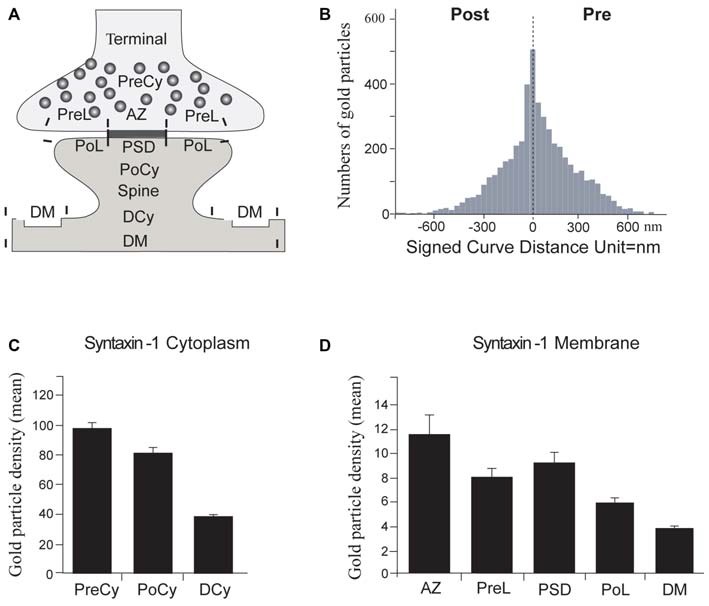
**Quantitation of synaptic syntaxin-1 immunogold labeling.** **(A)** Schematic drawing showing regions of interest in electron micrographs. PreCy, Presynaptic Cytoplasm; PreL, Presynaptic Lateral plasma membrane. AZ, Active Zone; PoCy, Postsynaptic Cytoplasm; PoL, Postsynaptic Lateral plasma membrane; PSD, PostSynaptic Density; DCy, Dendrite Cytoplasm; DM, Dendritic plasma Membrane. **(B)** Transverse histogram depicting the mean number of gold particles at every 30 nm distance from the center of the synaptic cleft, negative values are postsynaptic, positive values are presynaptic. The peak is over the synaptic plasma membranes. **(C)** Mean immunogold labeling over cytoplasmic regions of interest. **(D)** Mean immunogold labeling over plasma membrane regions of interest. Error bars show SEM, based on 180 micrographs of synapses/dendrites.

In order to assess the relative concentration of syntaxin-1 in different compartments of the synapse, and specifically to evaluate the degree of postsynaptic syntaxin-1 expression relative to the presynaptic expression, we quantified the mean number of immunogold particles over the different cytoplasmic and membrane compartments described in Figure 5A and in the “Materials and Methods” Section. With this analytical tool, we could then determine whether syntaxin-1 is present in the postsynaptic spine in comparable concentrations to those in the presynaptic terminal, as would be expected if it has a role in postsynaptic vesicle exocytosis.

We thus found syntaxin-1 to be significantly expressed in the postsynaptic spine, at concentrations only moderately lower than in the presynaptic terminal, but significantly higher than over corresponding dendritic compartments (Figures 5B–D). First, we calculated the mean number of immunogold particles as a function of the distance from a line drawn through the middle of the synaptic cleft (Figure 5B). This analysis showed that the highest concentrations of syntaxin-1 are clearly localized in the immediate vicinity (within 30 nm) to the two synaptic plasma membranes, as would be expected of a t-SNARE. Both pre- and postsynaptically, the concentration of syntaxin-1 tapers off abruptly in the cytoplasm interior to the synaptic plasma membranes.

Then we compared the labeling densities within the different cytoplasm and membrane compartments (Figures 5C,D). The postsynaptic plasma membrane at the PSD showed syntaxin-1 levels at around 80% of the AZ plasma membrane, but still about 45% more than dendritic membrane. Likewise, the PoCy labeling intensity was 80% of the corresponding presynaptic one, but double the concentration over dendritic cytoplasm (Figure 5C). Both presynaptic AZ labeling and postsynaptic plasma membrane labeling along the PSD were significantly stronger than their corresponding perisynaptic membrane labeling (Figure 5D).

### Postsynaptic NMDA Receptors Colocalizes with Syntaxin-1

**Figure 6:**
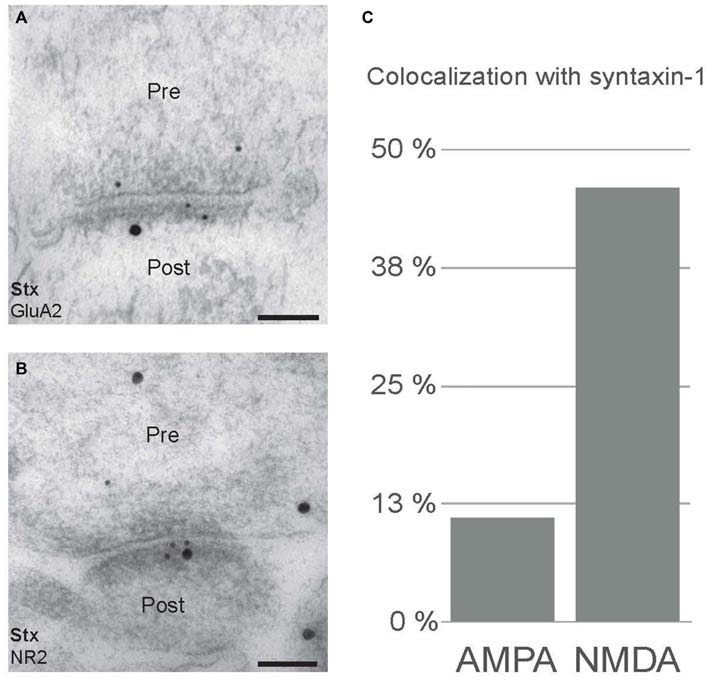
**Postsynaptic colocalization of syntaxin-1 and NMDA receptor subunits. (A)** Double immunogold labeling of syntaxin-1 (20 nm) and AMPA receptor subunit, GluA2 (10 nm). **(B)** Electron micrographs showing colocalization of syntaxin-1 (20 nm) and NMDA receptor subunit, NR2B (10 nm). Scale bars: **(A,B)** 100 nm. **(C)** Quantitative analysis of colocalization between syntaxin-1/GluA2 and syntaxin-1/NR2B in spines.

Postsynaptic SNARE proteins have been proposed to facilitate trafficking of glutamate receptors. In order to investigate this hypothesis, we performed immunogold double labeling with antibodies to syntaxin-1 and glutamate receptors (Figure 6). As expected, both syntaxin-1, and NMDA receptor (anti-NR2B; Figure 6B) and AMPA receptor (anti-GluA2; Figure 6A) immunogold labeling were found within postsynaptic spines. We then calculated the degree of colocalization, within the spines, of either NR2B or GluA2 with syntaxin-1 (Figure 6C). While 46% of NR2B-associated gold particles were localized in close proximity (distance ≤47 nm, see “Materials and Methods” Section) to syntaxin-1-associated gold particles, only 11% of GluA2-associated gold particles were localized closely to syntaxin-1-associated gold particles. Thus, we find evidence for association between syntaxin-1 and NR2B-containing NMDA receptors in postsynaptic spines, but much less between syntaxin-1 and GluA2-containing AMPA receptors.

### Control of Anti-Syntaxin-1 Labeling

The immunofluorescence staining of dissociated hippocampal cultures mentioned above were also used as control for our electron microscopy findings in intact hippocampus. The cultures were double labeled with anti-syntaxin-1 and either pre- or postsynaptic markers. Immunofluorescence labeling of the dissociated hippocampal cultures with anti-syntaxin-1 and anti-beta-tubulin (TUJ-1) showed syntaxin-1 positive synapses along the dendrites (Figures 4A–C). Labeling with anti-syntaxin-1 is punctate, as expected for a synaptic protein, although weak labeling of the dendritic profiles can also be seen. Double labeling with synaptophysin (P38) shows partial overlap between the proteins (Figures 4D–F), this confirms the expression of syntaxin-1 in presynaptic terminals. A similar pattern was observed in cultures double labeled with PSD-95 and syntaxin-1 (Figures 4G–I), i.e., both proteins are partially colocalized, confirming that syntaxin-1 is also present postsynaptically.

**Figure 7:**
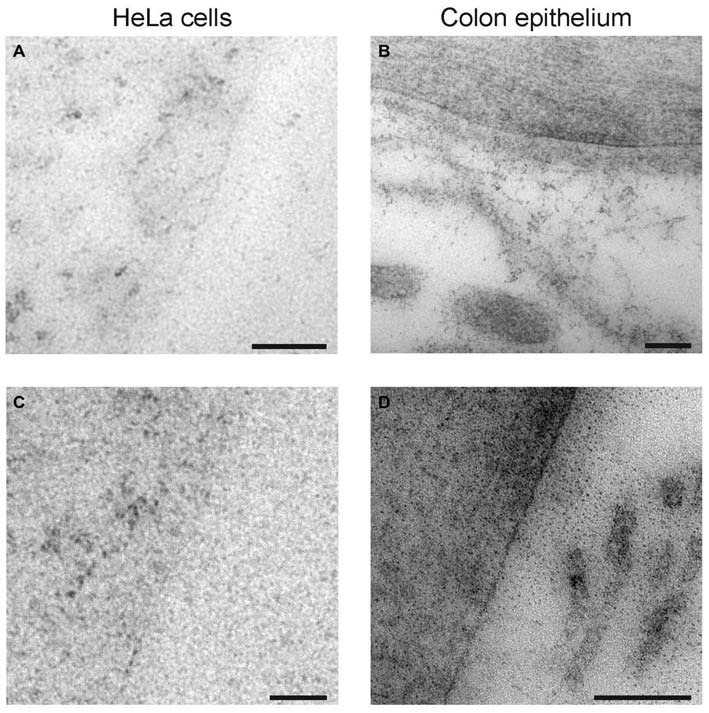
**Control of syntaxin-1 antibodies. (A,B)** Electron micrographs showing immunogold labeling of HeLa cell and colon epithelium with anti-syntaxin-1 (Millipore). **(C,D)** Electron micrographs showing immunogold labeling of HeLa cell and colon epithelium with anti-syntaxin-1 (Sigma). Scale bars: **(A–D)** 100 nm.

We also performed negative controls of the electron microscopy immunogold labeling. HeLa cells (Figures 7A,C) and rat colon epithelium cells (Figures 7B,D) were embedded and sectioned as the hippocampal tissue, before immunogold labeling with anti-syntaxin-1 antibodies. Very few gold particles were seen on these non-neuronal, non-syntaxin-1 expressing cells, as would be expected of highly specific anti-syntaxin-1 antibodies.

## Discussion

This is the first analysis of syntaxin-1 localization within a hippocampal synapse. Detailed quantitative analysis reveals that syntaxin-1 is not restricted to the presynaptic AZ. In fact, it is distributed throughout the pre- and postsynaptic elements, in the plasma membrane as well as in cytoplasmic regions. Though the highest concentrations are seen over the central synaptic plasma membranes, i.e., over both the AZ and the PSD, pre- and postsynaptic vesicles are also associated with syntaxin-1, as are the pre- and postsynaptic lateral membranes. Postsynaptic syntaxin-1 is often colocalized with NMDA receptors, but not with AMPA receptors, indicating that syntaxin-1 may be involved with trafficking of NMDA receptors and thus in regulating synaptic plasticity.

There are distinct variations in syntaxin-1 concentrations between different brain areas. This is evident both from regional western blot (Figure 1) and immunohistochemistry (Figure 2). The results of these two assays correspond reasonably well, with the highest concentrations seen in hippocampus and cerebellar cortex. In our recent study of VAMP2 in rat brain (Hussain and Davanger, 2015), we performed a similar regional analysis, showing that hippocampus and cerebellum seem to be the areas with the highest concentrations of this synaptic protein. Syntaxin-1 and VAMP2 are cognate SNAREs, where one would expect a similar distribution.

Few studies have investigated the relative density of synapses in different regions of gray matter in the rodent brain. Using the presynaptic vesicle protein synaptophysin as a probe, Saito et al. (1994) found indications that in young rats, the density of synapses is somewhat higher in hippocampus (5.54 μg/mg synaptophysin per tissue protein in homogenate) than in most areas of the cortex (about 4 μg/mg, though the frontal cortex was higher, at 6.09 μg/mg), corresponding with our relative higher concentrations of syntaxin-1 and VAMP2 in hippocampus than in cortex. On the other hand, they did find lower concentrations of synaptophysin in the cerebellum (2.88 μg/mg), which is different from our observation of almost as high syntaxin-1 concentrations in cerebellum as in hippocampus. When taking tissue samples from the cerebellum, it is difficult to separate gray matter form white matter, and the amount of white matter, probably devoid of syntaxin-1 and other synaptic proteins, may vary more from sample to sample. Furthermore, the concentrations of such proteins will probably vary between synapses from different brain areas in any case, due to functional differences, so that they cannot be used as an exact estimation of synapse density.

### Syntaxin-1 is Present at the Active Zone, Synaptic Presynaptic Vesicles, and Lateral Synaptic Plasma Membrane

So far, only a few studies have described ultrastructural localizations of syntaxin in the brain, focusing on the cerebellum. Koh et al. (1993) detected syntaxin-1 at high densities on plasma membrane and synaptic vesicles of presynaptic terminals on Purkinje cell dendrites, as well as on the plasma membrane of parallel fibers in the cerebellar molecular layer. In the granule cell layer, gold particles were also detected on the endoplasmic reticulum, nuclear membranes and the plasma membranes of granule cells. Miya et al. (1996) used immunoelectron microscopy to show that syntaxin-1 is localized on the extruding plasma membrane of the granule cells to form parallel fibers in the developing rat cerebellum. In 8-days-old rat, syntaxin-1 immunoreactivity appeared on the axons of parallel fibers and at the synapses.

There are no other studies providing quantitative information about the protein at ultrastructural levels of hippocampal synapses. In the present study, a total of 1440 profiles of Schaffer collateral synapses on pyramidal cells in the CA1 area were analyzed for syntaxin-1 labeling. Statistical analyses clarified the localization of the protein at the synapse. It is well known that SNARE proteins regulate exocytosis of presynaptic vesicles (Bennett and Scheller, 1994; Hussain and Davanger, 2011). Our results contribute to this knowledge by describing presynaptic syntaxin-1 localization in detail. Confirming that syntaxin-1 is a plasma membrane protein, we found strong concentrations of gold particles over the presynaptic AZ. However, significant levels of syntaxin-1 are also associated with presynaptic vesicles. These vesicles may either transport newly synthesized syntaxin-1 to the plasma membrane, or they may originate from endocytosis of plasma membrane after exocytosis.

Hagiwara et al. (2005) used sodium dodecyl sulfate-digested freeze-fracture replica labeling (SDS-FRL) to analyze quantitatively the distribution of syntaxin in the hippocampal CA3 area. They found that the density of immunoparticles for syntaxin was not significantly different between the AZ and the surrounding extrasynaptic plasma membrane. Our results, however, show that the perisynaptic lateral membrane contains 68% of the AZ syntaxin-1 level. This is still about double the concentration found over DM. Thus, our results are in line with the presynaptic AZ being a specialized membrane region for vesicle exocytosis, but both our results and those of Hagiwara et al. (2005) show that target SNARE proteins are not confined to the AZ, so that extrasynaptic exocytosis of transmitter cannot be excluded.

### Syntaxin-1 is Present at the PSD, Postsynaptic Vesicles, and Lateral Postsynaptic Plasma Membrane

Although there is abundant data on presynaptic SNARE protein localization and function, their possible postsynaptic expression and function has only recently begun to be elucidated (Jurado, 2014). Koh et al. (1993) embedded synaptosomes from rat cerebellum in agarose, and processed them with the pre-embedding protein A-gold technique for syntaxin-1 (HPC-1). Results showed that gold particles labeled cytoplasmic surfaces of the presynaptic membranes and synaptic vesicles. Later, others have provided indirect evidence for the postsynaptic expression of syntaxin-3 (Jurado et al., 2013) and syntaxin-4 (Kennedy et al., 2010) and their role in AMPA receptor insertion. We are the first to show that syntaxin-1 is located also postsynaptically. Syntaxin-1 immunoreactivity was found in postsynaptic plasma membrane and postsynaptic vesicles. The protein was not present in the same concentration as in the presynaptic terminals, but it was still more strongly expressed in the postsynaptic spines than in corresponding dendritic compartments. Also, postsynaptic localization patterns mimicked corresponding presynaptic ones. The protein was more concentrated over the PSD, significantly higher than over the postsynaptic lateral membrane. This may indicate a higher exocytotic activity at the PSD compared to the remaining postsynaptic plasma membrane. In fact, the transversal histogram of labeling density as function of the distance from the synaptic cleft (Figure 5B), shows a narrow, pointy curve with a peak at the synaptic plasma membranes, as expected from a tSNARE with only a moderate expression in the terminal and spine cytoplasms. A corresponding curve recently shown for the vSNARE VAMP2 (Hussain and Davanger, 2015) was wider and more bell-shaped, typical of a more cytoplasmic localization.

Our immunofluorescence labeling at the confocal microscopy level (Figure 4) supported our immunogold EM data, showing synaptic localizations of syntaxin-1 immunoreactivity. However, though both the synaptic markers synaptophysin and PSD-95 colocalized with syntaxin-1, it is not possible at this level of resolution to discern between pre- and postsynaptic sites. These two markers seem relatively unique in being found strictly pre- or postsynaptically, respectively. We utilized this in a biochemical assay (Figures 4J–L), where we separated the pre- and postsynaptic membranes before doing western blots, as has been done by others (Phillips et al., 2001). These blots then, confirm the immunogold data. Ideally, we should have validated the biochemical separation of the pre- and postsynaptic membranes with other, distinct pre- or postsynaptic proteins. However, this field is increasingly more complicated, with glutamate receptors being found also presynaptically, and many classical SNARE protein “presynaptic markers” now being seen also postsynaptically.

The observed postsynaptic vesicles were morphologically of similar type as the VAMP2-positive postsynaptic vesicles we have recently described (Hussain and Davanger, 2015). While VAMP2 is a known vSNARE, syntaxin-1 is a tSNARE, so there is little reason to assume that syntaxin-1 has a docking or fusion function on these vesicles. They could either be vesicles transporting syntaxin-1 to the synaptic plasma membranes, or they could be regular endocytotic vesicles originating from the plasma membrane where syntaxin-1 has not been separated out from the membrane before endocytosis.

While the observations were as expected for presynaptic compartments, this is the first time syntaxin-1 has been shown to be specifically expressed in the postsynaptic spines, with a localization pattern well compatible with a function in postsynaptic vesicle trafficking.

### Syntaxin-1 Colocalizes Closely with Postsynaptic NMDA Receptors

Other isoforms of syntaxin have been shown to play an important role in postsynaptic function. Syntaxin-4 was proposed to be the key syntaxin defining a site at which activity-dependent exocytosis occurs in dendritic spines during LTP (Kennedy et al., 2010). Later, Jurado et al. (2013) showed that syntaxin-3 is required for regulated AMPA receptor exocytosis during LTP. Although syntaxin-1 is not proposed as a candidate for postsynaptic receptor trafficking in these studies, our findings show that syntaxin-1 is significantly expressed in the postsynaptic compartment. This strongly indicates that different syntaxin isoforms coexist in the postsynaptic spine and may possibly participate in distinct trafficking pathways.

Lastly, in order to explore the hypothesis that syntaxin-1 plays a role in glutamate receptor trafficking in postsynaptic spines, we compared the degree of close colocalization, in the nanometer range, of both the NMDA receptor subunit NR2B, and the AMPA receptor subunit GluA2, with syntaxin-1. While the close colocalization of GluA2 with syntaxin-1 was low (11% of GluA2-attached gold particles were localized adjacent to syntaxin-1-attached gold particles), close colocalization of NR2B was high, at 46%. Thus, our results are compatible with Jurado et al. (2013) who did not find evidence for syntaxin-1 involvement in AMPA receptor trafficking. However, the significantly stronger association between NR2B and syntaxin-1A, combined with the high density of syntaxin-1 labeling over the PSD, points to the possibility that syntaxin-1 plays a role in exocytosis of NMDA receptors directly to the postsynaptic plasma membrane, at the PSD. Application of other methods, e.g., co-immunoprecipitation of the proteins or treatment of neurons with botulinum toxin, may further substantiate this possibility. Also, it would be highly interesting to investigate interactions between syntaxin-1 and other NMDA receptor subunits.

As mentioned above, syntaxin-1 binds to SNAP-25 and VAMP2, forming a core complex responsible for vesicle to plasma membrane fusion in the presynaptic terminal. We have recently found strong indications that, in postsynaptic spines, VAMP2 is involved with insertion of GluA1-containing AMPA receptors but not with GluA2-containing AMPA receptors (Hussain and Davanger, 2015). Our current results point to the possibility that syntaxin-1 is associated with NMDA receptors, but not, or much less, with GluA2-containing AMPA receptors in the same type of spines. Taken together, these observations are compatible with postsynaptic v-SNARE and t-SNARE proteins binding together with other cognate partners than in the presynaptic compartment. In line with this, others have shown that different VAMP-, SNAP- and syntaxin-proteins may bind together in many different combinations (Fasshauer et al., 1999; Yang et al., 1999), implying that different SNARE protein combinations may coexist within the same cell to regulate fusion events at different subcellular compartments (Jurado, 2014). Very likely, different sets or combinations of SNARE proteins may act together to regulate insertion of diverse receptors in or around postsynaptic spines.

## Conclusion

Our main result is that the target SNARE syntaxin-1 is expressed in hippocampal postsynaptic spines in concentrations that are only moderately lower than in presynaptic terminals, supporting the notion that it contributes to postsynaptic vesicle exocytosis. Detailed quantitative analysis of labeling densities within presynaptic terminals support the concept that vesicle exocytosis primarily occurs at the AZ, but may possibly also take place extrasynaptically, lateral to the AZ. Corresponding to our presynaptic findings, we conclude that the strongest postsynaptic syntaxin-1 concentrations are found at the PSD, where it colocalizes closely with NMDA receptors, but to a much lower degree with AMPA receptors. This is compatible with a route of NMDA receptor exocytosis directly into the synaptic plasma membrane. However, extrasynaptic exocytosis may possibly also occur, with syntaxin-1 being present also lateral to the PSD.

## Author Contributions

Conceived and designed the experiments: SH, SD. Electron microscopy experiments: SH. Quantification of the EM data: SH. Light microscopy experiments: HR. Western blot experiments: SH, DLE, TLS. Analyzed the data: SH. Wrote the article: SH, SD.

## Funding

The University of Oslo, and the European Union Projects QLG3-CT-2001-02089 (KARTRAP) and LSCHM-CT-2005-005320 (GRIPANNT) supported this work. The funders had no role in study design, data collection and analysis, decision to publish, or preparation of the manuscript.

## Conflict of Interest Statement

The authors declare that the research was conducted in the absence of any commercial or financial relationships that could be construed as a potential conflict of interest.

The reviewer Dr. Andras Bilkei-Gorzo and handling Editor, Dr. Ildikó Rácz, declared their shared affiliation, and the handling Editor states that the process nevertheless met the standards of a fair and objective review.

## References

[B1] Bajjalieh S. M. Scheller R. H. (1995). The biochemistry of neurotransmitter secretion. J. Biol. Chem. 270, 1971–1974. 10.1074/jbc.270.5.1971 7836421

[B2] Bennett M. K. Calakos N. Scheller R. H. (1992). Syntaxin: a synaptic protein implicated in docking of synaptic vesicles at presynaptic active zones. Science 257, 255–259. 10.1126/science.1321498 1321498

[B3] Bennett M. K. García-Arrarás J. E. Elferink L. A. Peterson K. Fleming A. M. Hazuka C. D.. (1993). The syntaxin family of vesicular transport receptors. Cell 74, 863–873. 10.1016/0092-8674(93)90466-4 7690687

[B4] Bennett M. K. Scheller R. H. (1994). Molecular correlates of synaptic vesicle docking and fusion. Curr. Opin. Neurobiol. 4, 324–329. 10.1016/0959-4388(94)90092-2 7919928

[B5] Besalduch N. Tomàs M. Santafé M. M. Garcia N. Tomàs J. Lanuza M. A. (2010). Synaptic activity-related classical protein kinase C isoform localization in the adult rat neuromuscular synapse. J. Comp. Neurol. 518, 211–228. 10.1002/cne.22220 19937712

[B6] Fasshauer D. Antonin W. Margittai M. Pabst S. Jahn R. (1999). Mixed and non-cognate SNARE complexes. Characterisation of assembly and biophysical properties. J. Biol. Chem. 274, 15440–15446. 10.1074/jbc.274.22.15440 10336434

[B7] Fonnum F. (1984). Glutamate: a neurotransmitter in mammalian brain. J. Neurochem. 42, 1–11. 10.1111/j.1471-4159.1984.tb09689.x 6139418

[B8] Gaisano H. Y. Ghai M. Malkus P. N. Sheu L. Bouquillon A. Bennett M. K.. (1996). Distinct cellular locations of the syntaxin family of proteins in rat pancreatic acinar cells. Mol. Biol. Cell 7, 2019–2027. 10.1091/mbc.7.12.2019 8970162 PMC276047

[B9] Hagiwara A. Fukazawa Y. Deguchi-Tawarada M. Ohtsuka T. Shigemoto R. (2005). Differential distribution of release-related proteins in the hippocampal CA3 area as revealed by freeze-fracture replica labeling. J. Comp. Neurol. 489, 195–216. 10.1002/cne.20633 15983999

[B10] Hanley J. G. (2008). AMPA receptor trafficking pathways and links to dendritic spine morphogenesis. Cell Adh. Migr. 2, 276–282. 10.4161/cam.2.4.6510 19262155 PMC2633691

[B33] Hasegawa H. Yang Z. Oltedal L. Davanger S. Hay J. C. (2004). Intramolecular protein-protein and protein-lipid interactions control the conformation and subcellular targeting of neuronal Ykt6. J. Cell. Sci. 117, 4495–4508. 10.1242/jcs.01314 15331663

[B11] Hayashi T. McMahon H. Yamasaki S. Binz T. Hata Y. Sudhof T. C.. (1994). Synaptic vesicle membrane fusion complex: action of clostridial neurotoxins on assembly. EMBO J. 13, 5051–5061. 7957071 10.1002/j.1460-2075.1994.tb06834.x PMC395451

[B12] Hussain S. Davanger S. (2011). The discovery of the soluble N-ethylmaleimide-sensitive factor attachment protein receptor complex and the molecular regulation of synaptic vesicle transmitter release: the 2010 Kavli prize in neuroscience. Neuroscience 190, 12–20. 10.1016/j.neuroscience.2011.05.057 21641968

[B13] Hussain S. Davanger S. (2015). Postsynaptic VAMP/synaptobrevin facilitates differential vesicle trafficking of GluA1 and GluA2 AMPA receptor subunits. PLoS One 10:e0140868. 10.1371/journal.pone.0140868 26488171 PMC4619507

[B14] Inoue A. Obata K. Akagawa K. (1992). Cloning and sequence analysis of cDNA for a neuronal cell membrane antigen, HPC-1. J. Biol. Chem. 267, 10613–10619. 1587842

[B15] Jahn R. Südhof T. C. (1994). Synaptic vesicles and exocytosis. Annu. Rev. Neurosci. 17, 219–246. 10.1146/annurev.neuro.17.1.219 8210174

[B16] Jurado S. (2014). The dendritic SNARE fusion machinery involved in AMPARs insertion during long-term potentiation. Front. Cell. Neurosci. 8:407. 10.3389/fncel.2014.00407 25565955 PMC4273633

[B17] Jurado S. Goswami D. Zhang Y. Molina A. J. Sudhof T. C. Malenka R. C. (2013). LTP requires a unique postsynaptic SNARE fusion machinery. Neuron 77, 542–558. 10.1016/j.neuron.2012.11.029 23395379 PMC3569727

[B18] Kennedy M. J. Davison I. G. Robinson C. G. Ehlers M. D. (2010). Syntaxin-4 defines a domain for activity-dependent exocytosis in dendritic spines. Cell 141, 524–535. 10.1016/j.cell.2010.02.042 20434989 PMC2874581

[B20] Koh S. Yamamoto A. Inoue A. Inoue Y. Akagawa K. Kawamura Y.. (1993). Immunoelectron microscopic localization of the HPC-1 antigen in rat cerebellum. J. Neurocytol. 22, 995–1005. 10.1007/bf01218356 8301329

[B21] Lang T. Jahn R. (2008). Core proteins of the secretory machinery. Handb. Exp. Pharmacol. 184, 107–127. 10.1007/978-3-540-74805-2_5 18064413

[B22] Low S. H. Chapin S. J. Weimbs T. Komuves L. G. Bennett M. K. Mostov K. E. (1996). Differential localization of syntaxin isoforms in polarized Madin-Darby canine kidney cells. Mol. Biol. Cell 7, 2007–2018. 10.1091/mbc.7.12.2007 8970161 PMC276046

[B23] Lu W. Man H. Ju W. Trimble W. S. MacDonald J. F. Wang Y. T. (2001). Activation of synaptic NMDA receptors induces membrane insertion of new AMPA receptors and LTP in cultured hippocampal neurons. Neuron 29, 243–254. 10.1016/s0896-6273(01)00194-5 11182095

[B34] Mathiisen T. M. Nagelhus E. A. Jouleh B. Torp T. Frydenlund D. S. Mylonakou M. N. (2006). “Postembedding immunogold cytochemistry of membrane molecules and amino acid transmitters in the central nervous system,” in Neuroanatomical Tract-Tracing 3: Molecules, Neurons, and Systems, eds Zaborszky L. Wouterlood F. G. Lanciego J. L. (New York: Springer), 72–108.

[B24] Miya F. Yamamoto A. Akagawa K. Kawamoto K. Tashiro Y. (1996). Localization of HPC-1/syntaxin 1 in developing rat cerebellar cortex. Cell Struct. Funct. 21, 525–532. 10.1247/csf.21.525 9078410

[B25] Nath A. R. Chen R. H. Stanley E. F. (2014). Cryoloading: introducing large molecules into live synaptosomes. Front. Cell. Neurosci. 8:4. 10.3389/fncel.2014.00004 24478628 PMC3899522

[B26] Phillips G. R. Huang J. K. Wang Y. Tanaka H. Shapiro L. Zhang W.. (2001). The presynaptic particle web: ultrastructure, composition, dissolution and reconstitution. Neuron 32, 63–77. 10.1016/S0896-6273(01)00450-0 11604139

[B27] Pozo K. Goda Y. (2010). Unraveling mechanisms of homeostatic synaptic plasticity. Neuron 66, 337–351. 10.1016/j.neuron.2010.04.028 20471348 PMC3021747

[B28] Rosenmund C. Stern-Bach Y. Stevens C. F. (1998). The tetrameric structure of a glutamate receptor channel. Science 280, 1596–1599. 10.1126/science.280.5369.1596 9616121

[B29] Saito S. Kobayashi S. Ohashi Y. Igarashi M. Komiya Y. Ando S. (1994). Decreased synaptic density in aged brains and its prevention by rearing under enriched environment as revealed by synaptophysin contents. J. Neurosci. Res. 39, 57–62. 10.1002/jnr.490390108 7807593

[B30] Söllner T. Bennett M. K. Whiteheart S. W. Scheller R. H. Rothman J. E. (1993). A protein assembly-disassembly pathway *in vitro* that may correspond to sequential steps of synaptic vesicle docking, activation and fusion. Cell 75, 409–418. 10.1016/0092-8674(93)90376-2 8221884

[B31] Volz F. Bock H. H. Gierthmuehlen M. Zentner J. Haas C. A. Freiman T. M. (2011). Stereologic estimation of hippocampal GluR2/3- and calretinin-immunoreactive hilar neurons (presumptive mossy cells) in two mouse models of temporal lobe epilepsy. Epilepsia 52, 1579–1589. 10.1111/j.1528-1167.2011.03086.x 21635231

[B32] Yang B. Gonzalez L. Jr. Prekeris R. Steegmaier M. Advani R. J. Scheller R. H. (1999). SNARE interactions are not selective. Implications for membrane fusion specificity. J. Biol. Chem. 274, 5649–5653. 10.1074/jbc.274.9.5649 10026182

